# Effect of Social Norm Messages on US Adults’ Antibiotic Use Intentions

**DOI:** 10.1093/ofid/ofag664

**Published:** 2026-10-14

**Authors:** Alistair Thorpe, Julia E Szymczak, Emely Luhan, Madeline C Farrell, William B Petty, Valerie M Vaughn, Angela Fagerlin, Rachael A Lee

**Affiliations:** Department of Population Health Sciences, Spencer Fox Eccles School of Medicine at University of Utah, Salt Lake City, Utah, USA; Division of Epidemiology, Department of Internal Medicine, Spencer Fox Eccles School of Medicine at University of Utah, Salt Lake City, Utah, USA; School of Psychology, Sport and Health Sciences, Faculty of Science and Health, University of Portsmouth, Portsmouth, UK; Department of Population Health Sciences, Spencer Fox Eccles School of Medicine at University of Utah, Salt Lake City, Utah, USA; Department of Population Health Sciences, Spencer Fox Eccles School of Medicine at University of Utah, Salt Lake City, Utah, USA; Department of Population Health Sciences, Spencer Fox Eccles School of Medicine at University of Utah, Salt Lake City, Utah, USA; Division of General Medicine, Department of Internal Medicine, Spencer Fox Eccles School of Medicine at University of Utah, Salt Lake City, Utah, USA; Salt Lake City VA Informatics Decision-Enhancement and Analytic Sciences Center for Innovation, Salt Lake City, Utah, USA; Department of Population Health Sciences, Spencer Fox Eccles School of Medicine at University of Utah, Salt Lake City, Utah, USA; Salt Lake City VA Informatics Decision-Enhancement and Analytic Sciences Center for Innovation, Salt Lake City, Utah, USA; Division of Infectious Diseases, Department of Medicine, UAB School of Medicine, Birmingham, Alabama, USA; Division of Infectious Diseases, Department of Medicine, Birmingham VA Medical Center, Birmingham, Alabama, USA

**Keywords:** antibiotic resistance, communication, public health, social norms

## Abstract

In this randomized survey of US adults (N = 972), public health messages explaining why antibiotics should be avoided for cold/flu symptoms reduced the desire for antibiotics for cold/flu symptoms. Social norm framing (62% do not vs 38% do) showed no significant effect on antibiotic intentions. Public communications should prioritize clear explanations.

## Graphical Abstract

**Graphical Abstract:**
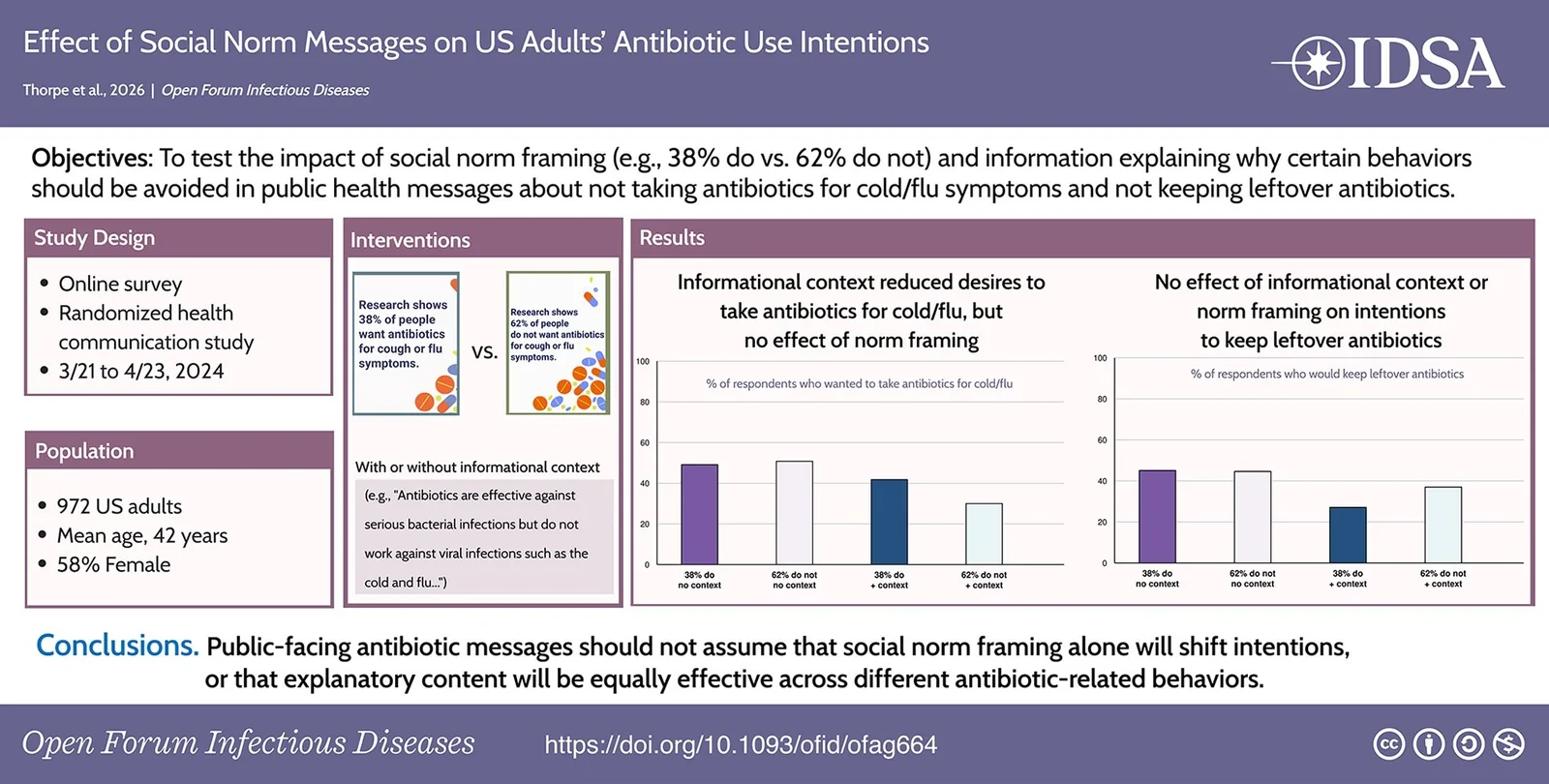
This graphical abstract is also available at Tidbit: https://tidbitapp.io/tidbits/effect-of-social-norm-messages-on-us-adults-antibiotic-use-intentions-bbd3ce69-a300-4ff0-9d58-c2530b9ec3f6

Unnecessary antibiotic use drives antimicrobial resistance (AMR)–associated infections, which contribute to millions of illnesses and deaths each year [1]. Recognition of the need for effective patient- and public-facing education about appropriate antibiotic use and AMR has grown rapidly over the past 2 decades. Patient- and public-facing education is now a core component of antibiotic stewardship programs [2] and public health campaigns [3]. As a result, identifying evidence-based strategies for communicating about antibiotics has emerged as an important research priority.

Educational messages explaining when antibiotics are necessary and the risks of unnecessary use can reduce clinically inappropriate desires for antibiotics but not for everyone and not completely [4, 5]. One of the most robust insights from social science and health communication research is that health decisions are shaped not only by knowledge but by social and psychological factors [6]. For instance, social norms (ie, perceptions of what other people do or approve of) strongly influence antibiotic-related beliefs and behaviors [6, 7], suggesting an opportunity for communication interventions [4]. However, less is known about how exactly to communicate specific antibiotic-related norms to patients and the public.

Moreover, while communicating norms has promoted antibiotic-related behavior change among clinicians [8] and among patients and the public in other health contexts (eg, reducing tobacco use), it can also “backfire” and make undesirable behaviors seem common, acceptable, or appealing [7, 9]. Evidence from medical decision-making research can help explain why messages about other people's health behaviors may have unintended effects. For instance, such messages may unintentionally promote undesirable behaviors by highlighting them as actions that people can take [4], emphasizing gains rather than losses (eg, “80% do” vs “20% do not”) [9, 10], or making the behavior seem socially typical (eg, “If others are doing it . . .”) [9]. This concern is particularly relevant when communicating about nonrecommended antibiotic behaviors, because messages describing what other people do may be interpreted differently depending how they are presented. However, there is currently an evidence gap to whether social norm messages about antibiotic use can promote public intentions to follow recommended antibiotics behaviors and whether the effects of such messages are influenced by message framing and contextual information about appropriate antibiotic use.

We aimed to address this gap by testing whether social norm framing of other people's antibiotic behaviors (38% of people do vs 62% do not) and the provision of contextual information explaining why the behaviors should be avoided (absent vs present) influence US adults’ willingness to engage in 2 nonrecommended antibiotic behaviors. Specifically, we focused on 2 common targets for public and patient education: wanting antibiotics for cold or flu symptoms and keeping leftover antibiotics for future use. We hypothesized that messages highlighting the percentage of people who engage in nonrecommended antibiotic behaviors (eg, “38% of people do”) would increase reported willingness to engage in those behaviors, whereas providing contextual information about appropriate antibiotic use (eg, “antibiotics are ineffective for cold/flu symptoms”) would decrease willingness.

## METHODS

From 21 March to 23 April 2024, we conducted an online survey with respondents recruited and compensated by Dynata, a commercial research company. Dynata emailed eligible adults a survey access link. The survey was deemed exempt by the University of Utah Institutional Review Board (IRB_00167676) with consent implied by opting to complete the survey. Eligibility was limited to US adults aged ≥18 years who could read English. For these analyses, adults aged ≥65 years were excluded as they were asked different age-specific questions for an unrelated study.

### Procedure

Respondents were first randomized to 1 of 2 study arms:

*Cold/flu arm:* shown only messages about antibiotics for cold or flu symptoms

*Keep leftovers arm:* shown only messages about keeping leftover antibiotics

Within both arms, respondents were randomized to see 1 of 4 public health messages about antibiotic use. As shown in Figure 1, all messages started with “Research shows”; then, they varied by social norm framing (62% of people do not vs 38% of people do), the study arm topic, and the presence (vs absence) of contextual information explaining why antibiotics should be avoided for cold and flu symptoms (eg, ineffective, lead to resistance) and leftovers should be disposed of properly. The percentages used in the social norm messages (62% vs 38%) were based on prior research indicating that approximately 38% of adults seeking care for upper respiratory infections want antibiotics [11]. For consistency, the same percentages were used for the “keep leftovers” messages.

**Figure 1.**
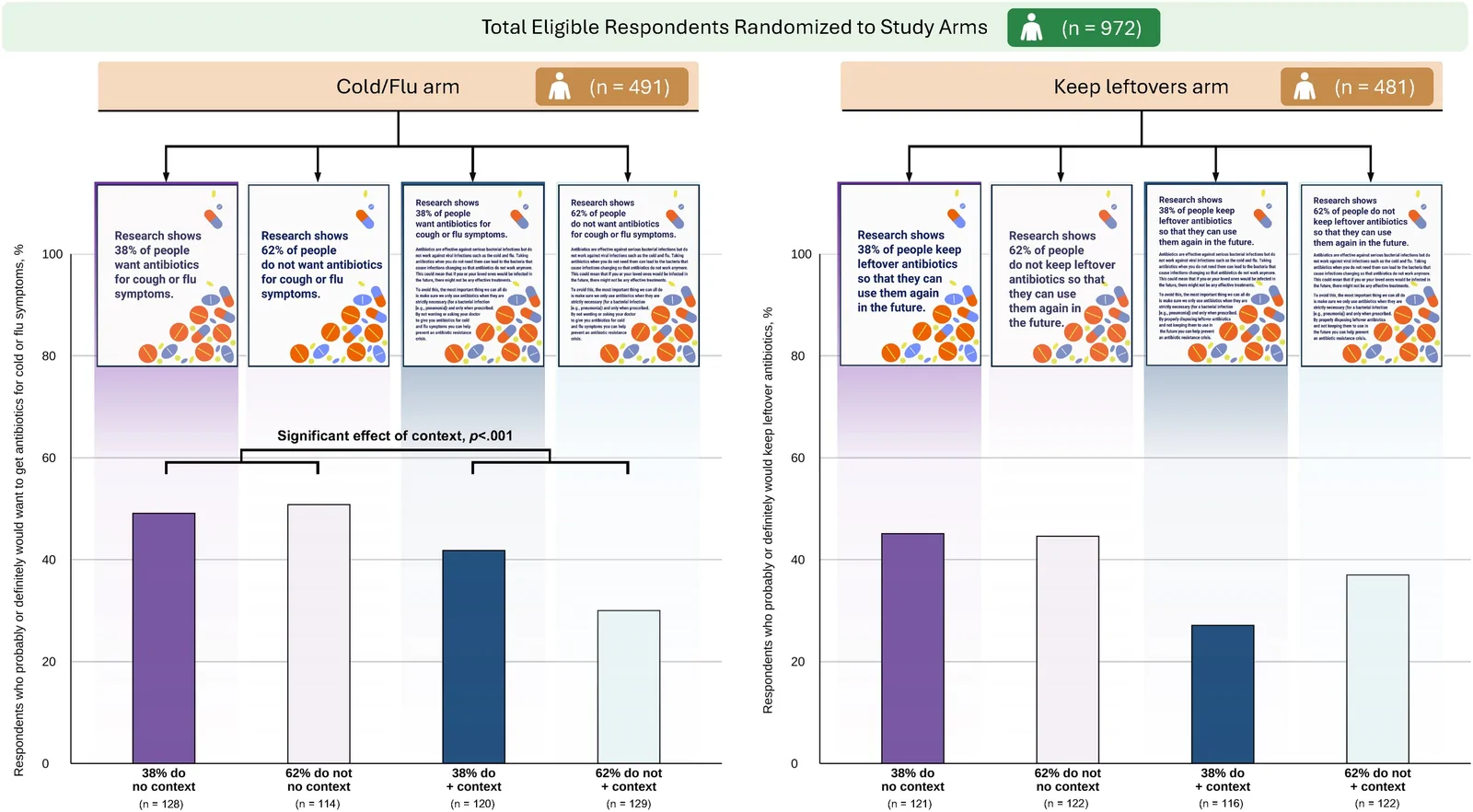
Primary outcomes by study arm and group assignment.

After being shown the message, participants responded to the primary outcome question tailored to their study arm. In the cold/flu arm, the outcome was wanting antibiotics for cold or flu symptoms (4-point response scale: 1 = definitely would not want to get antibiotics, 4 = definitely would want to get antibiotics). In the keep leftovers arm, the outcome was willingness to keep leftover antibiotics for future use (5-point response scale: 1 = I would never have leftovers, 5 = definitely would keep leftover antibiotics to use in the future). A full description of survey items and response scales for the present study along with the messages is shown in the Supplementary material.

### Analyses

We conducted 2 separate 2 × 2 factorial analyses of variance examining the effects of framing (38% do vs 62% do not) and contextual information (absent vs present) on wanting antibiotics and willingness to keep leftover antibiotics. Analyses were performed in RStudio 4.2.2 with statistical significance set at .05 (2-sided).

## RESULTS

Of the 1136 eligible respondents who started the survey, 1035 (91%) completed it. We then excluded 62 respondents whose free-text answers led us to question the validity of their data and 1 who finished in ≤5 minutes, resulting in a final sample of 972. The final sample had a mean age of 42 years and 58% identified as female (Table 1).

**Table 1.** Self-Reported Respondent Characteristics by Study Arm and Group Assignment

	Cold/Flu Study Arm (n = 491)				Keep Leftovers Study Arm (n = 481)				
	Group 1 (n = 128)	Group 2 (n = 114)	Group 3 (n = 120)	Group 4 (n = 129)	Group 1 (n = 121)	Group 2 (n = 122)	Group 3 (n = 116)	Group 4 (n = 122)	Overall (N = 972)
Contextual information	Absent		Present		Absent		Present		
Norm framing	62% do not	38% do	62% do not	38% do	62% do not	38% do	62% do not	38% do	
Age, y, mean (SD)	42.7 (14.6)	41.5 (13.7)	42.4 (12.8)	42.9 (13.2)	39.8 (12.8)	42.6 (12.4)	41.8 (12.9)	42.2 (12.3)	42.0 (13.1)
Age category, y									
18–33	43 (33.6)	42 (36.8)	39 (32.5)	42 (32.6)	45 (37.2)	32 (26.2)	39 (33.6)	34 (27.9)	316 (32.5)
34–49	31 (24.2)	34 (29.8)	38 (31.7)	40 (31.0)	40 (33.1)	50 (41.0)	41 (35.3)	47 (38.5)	321 (33.0)
50–64	54 (42.2)	38 (33.3)	43 (35.8)	47 (36.4)	36 (29.8)	40 (32.8)	36 (31.0)	41 (33.6)	335 (34.5)
Gender identity									
Female	49 (38.3)	53 (46.5)	48 (40.0)	61 (47.3)	41 (33.9)	48 (39.3)	48 (41.4)	51 (41.8)	560 (57.6)
Male	75 (58.6)	60 (52.6)	71 (59.2)	66 (51.2)	80 (66.1)	72 (59.0)	66 (56.9)	70 (57.4)	399 (41.0)
Any other identity	4 (3.1)	1 (0.9)	1 (0.8)	2 (1.6)	0 (0.0)	2 (1.6)	2 (1.7)	1 (0.8)	13 (1.3)
Racial/ethnic identity									
Hispanic	48 (37.5)	42 (36.8)	40 (33.3)	44 (34.1)	51 (42.1)	41 (33.6)	33 (28.4)	41 (33.6)	340 (35.0)
Non-Hispanic Black	50 (39.1)	39 (34.2)	43 (35.8)	48 (37.2)	43 (35.5)	42 (34.4)	43 (37.1)	44 (36.1)	352 (36.2)
Non-Hispanic White	24 (18.8)	22 (19.3)	20 (16.7)	23 (17.8)	14 (11.6)	24 (19.7)	25 (21.6)	24 (19.7)	176 (18.1)
Any other identity	6 (4.7)	11 (9.6)	17 (14.2)	14 (10.9)	13 (10.7)	15 (12.3)	15 (12.9)	12 (9.8)	103 (10.6)
Missing	0 (0.0)	0 (0.0)	0 (0.0)	0 (0.0)	0 (0.0)	0 (0.0)	0 (0.0)	1 (0.8)	1 (0.1)
US Census region									
Northeast	23 (18.0)	11 (9.6)	20 (16.7)	25 (19.4)	25 (20.7)	19 (15.6)	24 (20.7)	19 (15.6)	166 (17.1)
Midwest	20 (15.6)	20 (17.5)	15 (12.5)	27 (20.9)	22 (18.2)	15 (12.3)	19 (16.4)	22 (18.0)	160 (16.5)
South	66 (51.6)	53 (46.5)	55 (45.8)	55 (42.6)	47 (38.8)	59 (48.4)	49 (42.2)	57 (46.7)	441 (45.4)
West	18 (14.1)	28 (24.6)	30 (25.0)	22 (17.1)	26 (21.5)	29 (23.8)	24 (20.7)	24 (19.7)	201 (20.7)
Missing	1 (0.8)	2 (1.8)	0 (0.0)	0 (0.0)	1 (0.8)	0 (0.0)	0 (0.0)	0 (0.0)	4 (0.4)
Residence^a^									
Rural	30 (23.4)	18 (15.8)	35 (29.2)	31 (24.0)	36 (29.8)	31 (25.4)	29 (25.0)	29 (23.8)	239 (24.6)
Suburban	56 (43.8)	56 (49.1)	48 (40.0)	60 (46.5)	49 (40.5)	51 (41.8)	48 (41.4)	53 (43.4)	421 (43.3)
Urban	41 (32.0)	40 (35.1)	37 (30.8)	38 (29.5)	36 (29.8)	40 (32.8)	39 (33.6)	39 (32.0)	310 (31.9)
Missing	1 (0.8)	0 (0.0)	0 (0.0)	0 (0.0)	0 (0.0)	0 (0.0)	0 (0.0)	1 (0.8)	2 (0.2)
Educational attainment									
High school or less	30 (23.4)	36 (31.6)	29 (24.2)	35 (27.1)	29 (24.0)	33 (27.0)	24 (20.7)	28 (23.0)	244 (25.1)
Some college or trade	41 (32.0)	38 (33.3)	34 (28.3)	41 (31.8)	43 (35.5)	36 (29.5)	45 (38.8)	38 (31.1)	316 (32.5)
≥Bachelor	56 (43.8)	40 (35.1)	56 (46.7)	53 (41.1)	49 (40.5)	53 (43.4)	47 (40.5)	56 (45.9)	410 (42.2)
Missing	1 (0.8)	0 (0.0)	1 (0.8)	0 (0.0)	0 (0.0)	0 (0.0)	0 (0.0)	0 (0.0)	2 (0.2)

Data are presented as No. (%) of respondents unless noted otherwise.

^a^Categories were defined as follows: rural = rural, other, small (<100 000); suburban = suburban near large city; urban = midsized city (100 000–1 million), large city (>1 million).

### Cold/Flu Arm

Overall, 211 of 491 respondents (43.0%) said that they probably or definitely would want to get antibiotics for cold or flu symptoms. Respondents who received the public health message with contextual information explaining why antibiotics should be avoided for cold or flu symptoms were significantly less likely to want antibiotics (90 of 249, 36.1%) than those who did not receive this information (121 of 242, 50.0%; *P* < .001). We found no significant effect of social norm framing (*P* = .16) and no significant interaction between contextual information and framing (*P* = .08).

### Keep Leftovers Arm

Overall, 185 of 481 respondents (38.5%) said that they probably or definitely would keep leftover antibiotics so that they could use them again in the future. We found no significant differences based on (1) whether respondents received contextual information about why it is important to dispose of antibiotics properly (*P* = .05) or (2) social norm framing (*P* = .13), as well as no interaction between contextual information and framing (*P* = .71).

## DISCUSSION

This online randomized survey experiment studied whether social norm framing (62% of people do not vs 38% of people do) and contextual information in public health messages influence US adults’ willingness to engage in clinically nonrecommended antibiotic behaviors. We examined these messaging strategies across 2 common targets for antibiotic education: wanting antibiotics for cold or flu symptoms and keeping leftover antibiotics. Contrary to our expectations, we found no effect of social norm framing on desires to get antibiotics for cold or flu symptoms or willingness to keep leftover antibiotics. Consistent with prior research, contextual information about appropriate antibiotic use decreased reported desires to get antibiotics for cold or flu symptoms [4, 5]. However, this effect did not extend to messages about keeping leftover antibiotics. One explanation is that respondents may be less familiar with messages discouraging keeping leftover antibiotics, which may have reduced their effectiveness [12]. Future studies might explore whether these messages may be more effective after repeated exposure or in countries where not keeping leftovers has been more widely emphasized [13]. Future work should also consider further investigating potentially modifiable factors underlying desires to keep leftover antibiotics [12]. It is important to note that cultural and practical factors (eg, medication costs and limited access to care) may also motivate people to keep leftover antibiotics and may limit the impact of public-facing messages. Given the limited effects of social norm framing observed in this study, future research might consider examining more novel, participatory approaches to public engagement about antibiotics that may resonate more strongly (eg, evaluating the impact of arts-based strategies such as the AMR Clinical Reference Center's annual senryu poetry contest on antibiotics and resistant bacteria).

### Limitations

Findings may not generalize to groups who may have been underrepresented within our sample (eg, those less able or willing to complete an online English-language survey). The cross-sectional design limits our ability to assess whether message effects differ with repeated exposure or emerge over longer time horizons. An impact of social norm messages may also depend on factors that we did not address, including the message source (eg, health care provider vs health institution) [14], delivery contexts (eg, poster in a provider’s office vs social media) [15], the use of verbal terms instead of numerical expressions, and differing norm magnitudes [9]. For example, we examined a single comparison (62% vs 38%), and it is possible that more pronounced majority-vs-minority comparisons (eg, 95% vs 5%) may have produced different effects [9]. Additionally, this study was not designed to assess whether any effects of the messages may have differed across respondent characteristics (eg, their regional, educational attainment, and urbanicity distributions). Examining potential interactions between experimental conditions and respondent characteristics represents an important direction for future research.

## CONCLUSIONS

In this study, social norm framing that emphasized the proportion of people who do not engage in nonrecommended antibiotic behaviors, as compared with the proportion who do, did not affect US adults’ reported desire for antibiotics for cold or flu symptoms or willingness to keep leftover antibiotics. Contextual information explaining why these behaviors should be avoided reduced desire for antibiotics for cold or flu symptoms but did not affect willingness to keep leftover antibiotics. Public-facing antibiotic messages should therefore not assume that social norm framing alone will shift intentions or that explanatory content will be equally effective across different antibiotic-related behaviors. Future work should examine other risk communication strategies to identify how best to communicate the risks of unnecessary antibiotic use and saving leftover antibiotics.

## Supplementary Material

ofag664_Supplementary_Data

## Supplementary Data

Supplementary materials are available at *Open Forum Infectious Diseases* online. Consisting of data provided by the authors to benefit the reader, the posted materials are not copyedited and are the sole responsibility of the authors, so questions or comments should be addressed to the corresponding author.
