# Supplementary material for: Effect of Social Norm Messages on US Adults’ Antibiotic Use Intentions
Source: Open Forum Infect Dis. 2026 Oct 14;13(10):ofag664. doi: 10.1093/ofid/ofag664 (PMC13684398; doi:10.1093/ofid/ofag664)
Supplement: Supplementary file 1 [file ofag664_supplementary_data.docx]

**Title:** Effect of Social Norm Messages on US Adults’ Antibiotic Use Intentions

**Short Title:** Social Norm Messages for Antibiotic Use

**Authors:** Alistair Thorpe PhD^1^, Julia E. Szymczak PhD^2^, Emely Luhan BSc*, Madeline C. Farrell MSc^1^, William B. Petty BSc^1^, Valerie M. Vaughn MD, MSc^3,4,1^, Angela Fagerlin PhD^1,4^ Rachael A. Lee MD, MSPH^5,6^

1) Department of Population Health Sciences, Spencer Fox Eccles School of Medicine at University of Utah, Salt Lake City, UT, USA
2) Department of Internal Medicine, Division of Epidemiology, Spencer Fox Eccles School of Medicine at University of Utah, Salt Lake City, UT, USA
3) Department of Internal Medicine, Division of General Medicine, Spencer Fox Eccles School of Medicine at University of Utah, Salt Lake City, UT, USA
4) Salt Lake City VA Informatics Decision-Enhancement and Analytic Sciences (IDEAS) Center for Innovation, Salt Lake City, UT, USA
5) Department of Medicine, Division of Infectious Diseases, UAB School of Medicine, Birmingham, AL, USA
6) Department of Medicine, Division of Infectious Diseases, Birmingham VA Medical Center, Birmingham, AL, USA

*	No current affiliation

**Corresponding author**: Alistair Thorpe PhD, Department of Population Health Sciences, Spencer Fox Eccles School of Medicine at University of Utah, 295 Chipeta Way, Salt Lake City, UT, USA 84108 | (801) 587-0742 ([alistair.thorpe@hsc.utah.edu](mailto:alistair.thorpe@hsc.utah.edu))

**Supplement description**

This supplement contains the full description of survey items for the present study.

**Survey items**

**Study information:**

We invite you to participate in a research study about decisions you make regarding your health. In this study, you will be asked some questions about your personal opinions, previous decisions, and experiences regarding antibiotics, common infections, and COVID-19. We will also ask some questions about yourself. If you agree to participate, we would like you to answer the questions on the following screens.

It will take approximately 15 to 20 minutes to complete this survey. You are free to skip any questions that you prefer not to answer. Compensation for participation will be provided in accordance with your panel agreement.

Every effort will be made to protect your privacy and confidentiality. We will not collect your name or any identifying information about you. Your participation will be completely anonymous and it will not be possible to link you to your responses.

This study is not designed to benefit you directly. You have a choice about being in this study. You do not have to be in this study if you do not want to be.

The data we collect will be used for this study but may also be important for future research. Your data may be used for future research or distributed to other researchers for future study without additional consent if information that identifies you is removed from the data.

Taking part in this research study is completely voluntary. If you do not wish to participate in this study, simply click in the corner to close the web browser window.

You may have questions about your rights as someone in this study. If you have questions, you can call the University of Utah Institutional Review Board (the responsible Institutional Review Board) at 801-581-3655. Questions or concerns about this study or interest in the final results may be directed to Dr. Valerie Vaughn at valerie.vaughn@hsc.utah.edu. Thank you for taking part in this study.

**Demographics**:

What is your age?

How would you describe your gender identity?

- Female (1)
- Male (2)
- Transgender woman/Transwoman (3)
- Transgender man/Transman (4)
- Non-Binary/Third gender (5)
- Prefer not to say (6)
- Other please specify (7)

How would you describe your ethnic group or background?

(Please select all that apply)

- American Indian or Alaskan Native (1)
- Asian or Asian American (2)
- Black or African American (3)
- Native Hawaiian or other Pacific Islander (4)
- White or European American (5)
- Other please specify (6)

Are you Hispanic or Latino/a or Latinx?

- No (1)
- Yes (2)

What state do you live in?

↓ Alabama (1)…Wyoming (52)

What is the highest level of schooling you have completed? (Check one)

- None (1)
- Elementary school (2)
- Some high school but no diploma (3)
- High school (Diploma or GED) (4)
- Some college, but no degree (5)
- Trade school (6)
- Bachelor’s degree (BS, BA, etc.) (7)
- Master’s degree (MA, MPH, etc.) (8)
- Doctoral/Professional degree (PhD, MD, etc.) (9)

How would you best describe the place where you live?

- Rural (1)
- Small city, e.g. less than 100,000 people (2)
- Suburban, near a large city (3)
- Mid-sized city, 100,000 to 1 million people (4)
- Large city, more than 1 million (5)
- Other, please specify (6)

Cold/Flu study arm: Public Health Messages

|  |  |
| --- | --- |
|  |  |
|  |  |

Would you want to get antibiotics for cold or flu symptoms?

- Definitely WOULD NOT want to get antibiotics (1)
- Probably WOULD NOT want to get antibiotics (2)
- Probably WOULD want to get antibiotics (3)
- Definitely WOULD want to get antibiotics (4)

Keep leftovers study arm: Public Health Messages

|  |  |
| --- | --- |
|  |  |
|  |  |

Would you ever keep any leftover antibiotics you have so that you could use them again in the future?

- I would never have any leftover antibiotics (i.e., I always finish the full course)” (1)
- Definitely WOULD NOT keep leftover antibiotics to use them again in the future (2)
- Probably WOULD NOT keep leftover antibiotics to use them again in the future (3)
- Probably WOULD keep leftover antibiotics to use them again in the future (4)
- Definitely WOULD keep leftover antibiotics to use them again in the future (5)

**Debrief:** Thank you for your participation! You are now finished with this survey. In this study, we are interested in understanding how to communicate about the risks of antibiotic resistance and people’s attitudes and understanding of antibiotics. We greatly appreciate all your responses! We are learning a lot about these topics, and we hope to share our findings broadly soon.

For accurate, up-to-date information please see the following CDC websites:

- Antibiotic resistance: <https://www.cdc.gov/drugresistance/index.html>
- Patient resources about antibiotics: <https://www.cdc.gov/antibiotic-use/materials-references/index.html>
- COVID-19: <https://www.cdc.gov/coronavirus/2019-ncov/index.html>

Thank you for participating in this survey.
